# Flavor release and stability comparison between nano and conventional emulsion as influenced by saliva

**DOI:** 10.1007/s13197-022-05534-w

**Published:** 2022-06-26

**Authors:** Oluwatofunmi Sanni, Catriona M. Lakemond, Ofir Benjamin

**Affiliations:** 1 Food Science Department, DN Upper Galilee, Tel Hai College, 12210 Upper Galilee, Israel; 2 Food Quality and Design, Wageningen University and Research, 6700 AA Wageningen, The Netherlands

**Keywords:** Nano-emulsion, High-pressure homogenization, Partition coefficient, Flavour release, Saliva

## Abstract

Flavour release and emulsion stability depend on volatile organic compounds' environmental conditions, food microstructure, and physicochemical properties. The effect of pH (3.5 vs 7.0) and saliva addition on stability and flavour release from nano and conventional emulsions was investigated using particle size, charge and Lumisizer measurments. Larger particle sizes were observed at lower pressures and in saliva-containing emulsions. At 1700 bar, nano-emulsions (below 150 nm) were created at pH 3.5 and 7.0 including saliva-containing emulsions. As was clear from the creaming velocity measurements, saliva addition decreased the emulsion stability by reducing particle charges and increased viscosity by more than 50%, especially when prepared at pH 3.5 closer to the isoelectric point of the used emulsifier β-lactoglobulin (pH 5.2). (5.2). Flavour release from emulsions was measured at equilibrium using a phase ratio variation to determine partition coefficients and dynamically using an electronic nose. Partition coefficients of the flavour compounds for most conditions were two to four times lower in emulsions prepared at pH 7.0 than at pH 3.5 and in emulsions without saliva. Emulsions prepared with higher pressures showed stronger flavor release rates, while additional salvia dropped the release rate for ethyl acetate at pH 3.5. The physicochemical properties of flavour compounds, saliva addition and pH of emulsions influenced flavour release more than homogenization pressures. The potential in using nano-emulsions in food applications an be attributed higher stability and enhanced flavor release.

## Introduction

Flavour perception has a notable impact on food choice and acceptability. During the consumption of food, flavour perception occurs when odour-active molecules are released from the food matrix and bind to receptors in the nasal cavity's olfactory epithelium (Mao et al. 2017). Oral processing induces physicochemical changes in the mouth that could alter the food structure, consequently affecting the release of volatile organic compounds (VOCs). In the mouth, factors such as pH and temperature differences between the product matrix and the mouth, chewing, tongue compression, and mixing with saliva can potentially affect flavour release. Saliva serves as a solvent in the mouth and binds or releases flavour compounds to the retronasal cavity. It influences flavour release through solubilisation and chemical interaction between saliva components and flavour molecules (Benjamin et al. 2013). The impact of saliva is essential in food emulsions since these systems possess multiple phases, and saliva can alter their distribution, affecting both the stability and release of flavour compounds in the emulsions. Saliva affects the partitioning of VOCs between the food matrix and gas-phase via dilution thus, influencing the dissolution and transport of flavour compounds to the receptors. Salivary mucins can also bind to VOCs and control their release (Canon and Neyraud 2017). Due to buffering capabilities of saliva, the pH of the consumed emulsion product can be increased. The closer the pH of an emulsion system is to the isoelectric point (pI) of the emulsifier used, the less electrostatic repulsion exists and the susceptibility to destabilization increases. Structural instabilities such as coalescence and flocculation have been reported in emulsions on the addition of saliva (Benjamin et al. 2013; Silletti et al. 2007).

Flavour release is also influenced by factors such as the nature of the VOCs, environmental conditions, food microstructure, and flavour affinity towards the food matrix (Paravisini and Guichard 2017). The properties of VOCs, such as high volatility and hydrophobicity, low solubility, and susceptibility to oxidation, limit their application in food formulations (Saffarionpour 2019). Encapsulation systems such as nano-emulsions, multilayer emulsions, and nanogels, amongst others, have been designed to improve stability, solubility, and controlled release of volatile flavour molecules to overcome these limitations (Kwan and Davidov-Pardo 2018; Shin et al. 2015).

Nano-emulsions are increasingly used as flavour encapsulation systems in the food and beverage industry (Goindi et al. 2016). Compared to conventional emulsions, nano-emulsions have smaller droplets sizes ranging between 20 and 250 nm (Liang et al. 2012; Yang et al. 2018). High-pressure homogenization (HPH) reduces droplet sizes of coarse emulsions, initially prepared with high shear/speed mixers, into nanoscale droplets. The coarse emulsion is fed into the narrow valve of the homogenizer where intense shear and turbulence create disruptive forces that shatter the droplets into smaller sizes, which further decrease with the increased number of homogenization cycles (Liu et al. 2019; Saffarionpour 2019). Previous authors have reported producing stable nano-emulsions with sizes smaller than 200 nm using HPH at pressures between 1000 and 2000 bar (Kwan and Davidov-Pardo 2018; Liang et al. 2012). Nano-emulsions, including high-pressure homogenized nano-emulsions, provide benefits such as improved emulsion homogeneity, stability against oxidation, coalescence and creaming, enhanced solubility and flavour retention, and controlled VOC release due to their small droplet sizes (Kwan and Davidov-Pardo 2018; Lane et al. 2020; Saffarionpour 2019).

Flavour release from a nano-emulsion system depends on volatility, partitioning, and mass transfer of the VOCs through the oil phase, interface, aqueous phase, and headspace. Partition coefficient (K) is the ratio of the flavour compound concentration in the gas phase to the concentration in the food product. Nano-emulsion properties such as particle size distribution, pH and viscosity can influence flavour compound partitioning and diffusion, consequently resulting in a flavour release (Mao et al. 2017). Emulsion destabilizations such as creaming, and coalescence alter the system's microstructure and thus affect flavour diffusion and release.

Previous research has focused on flavour release in conventional emulsions (Benjamin et al. 2013; Mao et al. 2017), and the characterization of physical properties and stability of various high-pressure homogenized nano-emulsion systems (Kwan and Davidov-Pardo 2018; Liang et al. 2012; Yang et al. 2018). Other studies found that smaller droplets with larger interfacial areas absorb more flavour compounds in emulsions, reducing air–liquid partition coefficients and slower flavour release (Meynier et al. 2005; van Ruth et al. 2002). Some authors however found that because of a larger interfacial area and shorter travel distance from the droplet centre to the interface, flavour molecules were released faster in emulsions with finer droplets (Mao et al. 2017). There is a dearth of knowledge on the flavour release and stability of high-pressure homogenized nano-emulsions under oral processing conditions such as saliva and body temperature.

Therefore, this study aims to investigate the stability and release of volatile organic compounds in high-pressure homogenized nano-emulsions as influenced by pH, homogenization pressure, and saliva. For comparison, conventional emulsions were produced using low homogenization pressures.

## Materials and methods

Emulsions were prepared using canola oil purchased from a local supermarket. Davisco Foods International, Inc. (Eden Prairie, Minnesota, USA) provided β-lactoglobulin with a purity above 95%. Sodium azide and phosphoric acid were obtained from Daejung Chemicals & Metals Co., Ltd. (Siheung, Gyeonggi, Republic of Korea). Four volatile organic compounds (VOCs) of different physicochemical properties (Table 1) were used: ethyl acetate, 1-penten-3-one, trans-2-hexenal and nonanal (Sigma-Aldrich, St. Louis, Missouri, USA). The purity of all VOCs was above 95%.

**Table 1:** Physicochemical properties of selected volatile organic compounds (VOCs)

Volatile flavour compound	Chemical formula	Boiling point (°C)	Log P^a^	Vapour pressure (mmHg) 25 °C	Water solubility (g/L) 25 °C
Ethyl acetate	C_4_H_8_O_2_	77	0.7	73	88
1-Penten-3-one	C_5_H_8_O	103	1.0	31	20
Trans-2-hexenal	C_6_H_10_O	146	1.8	4.6	5.3
Nonanal	C_9_H_18_O	191	3.5	0.5	0.1

Lide (2005) and TGSC (2009)

^a^Partition coefficient between octanol and water

### Nano-emulsion preparation

Emulsion systems with pH values of 3.5 and 7.0 were prepared to simulate food emulsions in acidic (e.g., fruit shakes, yoghurts) and neutral (e.g., milk drinks) conditions, respectively. Oil-in-water nano-emulsions were prepared using high-pressure homogenization. Coarse emulsions were made by mixing 5 mM phosphate buffer (pH 7), 3% canola oil and 1% β-lactoglobulin with a high-speed disperser (IKA, T18 digital ULTRA-TURRAX, Staufen, Germany) at 4400 rpm for 30 s until homogeneous. The emulsions were then passed through a high-pressure homogenizer (APV-2000, SPX flow technology Rosista GmbH, Unna, Germany) five times at pressures 100, 500, 1000 and 1700 bar. Following homogenization, 0.02% (w/w) sodium azide was added as a preservative, and the emulsions were stored at 4 °C in a laboratory refrigerator. Emulsions with a pH of 3.5 were prepared by gradually adding up to 20 mL of 0.2 M phosphoric acid to the emulsions and mixed. The pH was further adjusted with 0.1 M NaOH until it reached 3.5. Emulsion samples were measured within two weeks after preparation.

### Saliva collection

Saliva was obtained from 14 subjects (4 males and 10 females) between 26 and 46 years old, to simulate salivation during oral processing. The samples were collected by spitting in the morning after the subjects had rinsed their mouths. For at least an hour before the collection, subjects consumed only water and the first milliliter of saliva was discarded. The saliva was kept in an ice bath until the collection was completed. The pH of the saliva samples 7.6 ± 0.6, and conductivity of 2.5 ± 0.8 mS/cm before being frozen at -50 °C and thawed for several minutes before each test. During saliva-containing sample analyses, a 1:2.5 emulsion-and-saliva mixture was prepared.

### Particle size distribution

The particle size distribution was measured using the laser scattering technique using a particle size analyzer (Mastersizer 3000, Malvern Instruments Ltd, Worcestershire, UK). Approximately 0.1–1 mL of the emulsion (without and with saliva) was added to a 5 mM phosphate buffer (buffer of pH 5.6 for emulsion at pH 3.5 or of pH 7.0 for emulsion at pH 7.0). The instrument software was programmed with a refractive index of 1.47 and an absorbance value of 0.001. The surface area mean (d_3,2_) and volume-weighted mean (d_4,3_) diameters were reported.

### Particle charge

Surface charges of the emulsions were measured using a particle charge detector (Mütek™ PCD-05, BTG Instruments GmbH, Weßling, Germany). The emulsions were diluted tenfold in 5 mM phosphate buffer (pH 3.5 or 7.0) and mixed gently with saliva at 1:2.5 ratios. Emulsions were titrated with 10 mM poly-diallyl dimethylammonium chloride (PolyDADMAC) cationic solution or 10 mM polyethylene sulfonate (PES-Na) anionic solution until they reached a potential close to 0 mV.

### Viscosity

The apparent viscosity (mPas) of emulsion samples was measured at a fixed shear rate of 100/s using a viscometer (RheolabQC, Anton Paar GmbH, Graz, Austria). Emulsions were mixed and heated in a water bath to a temperature of approximately 40 °C. During the test, about 12 mL of the emulsion was introduced into a double gap sample cell (DG42) that was maintained at a temperature of 40 °C in the water bath.

### Creaming velocity

Creaming velocity, which is the linear integration of light transmission versus time at a constant speed, was used to determine emulsion stability. The creaming velocity was measured using an optical analytical centrifuge, LUMiSizer dispersion analyser (LUM GmBH, Berlin, Germany). The analysis was conducted at 37 °C and 4000 rpm for 2 h at 30 s intervals.

### Static headspace analysis

The concentrations of VOCs in the headspace were determined by gas chromatography-mass spectrometry (GC–MS) under thermodynamic equilibrium. The volatile flavour compounds (ethyl acetate, nonanal, 1-penten-3-one and trans-2-hexenal) were prepared in propylene glycol. A mixture of the compounds (0.004% v/v each) was added to 3 mL emulsion and vortexed. To get a linear curve from which the partition coefficient for each flavour compound would be calculated, four volumes of samples (20, 40, 100, 500 µL) were transferred to 20 mL headspace vials, agitated for 30 min at 250 rpm to ensure equilibrium, and incubated at 35 °C in an automated headspace sampler. A single headspace sample (1 mL) was drawn from each vial and injected into the GC using a gas-tight syringe. The GC (6890 N, Agilent Technologies, Palo Alto, CA, USA) had a 250 °C inlet port and a DB-WAX capillary column (60 m length, 0.25 mm diameter, and 0.25 µm film thickness; J&W DB-WAX, Agilent Technologies, Palo Alto, CA, USA). The oven was programmed to heat from 40 °C to 250 °C at a rate of 5 °C/min. Helium was used as the carrier gas at a flow rate of 1.5 mL/min. The MS was operated in electron impact mode (EI/MS) at 70 eV and scanned in a range of m/z 40–450. For quantification, a calibration curve was plotted using calculated peak areas from the GC analysis against four known concentrations of each VOC. Linear regression coefficients were calculated, and VOC partition coefficients (K) between the gas and matrix phases were determined by the phase ratio variation (PRV) method using Eqs. 1 and 2:

1 $$ \frac{1}{A} = \frac{K}{{f_{i} *C_{s} }} + \frac{1}{{f_{i} *C_{s} }}*\beta $$

2 $$ \frac{1}{A} = b + a*\beta $$

where A is the peak area from the GC, C_s_ is the concentration of VOC in the sample phase, f_i_ is a proportionality factor, and β is the phase volume ratio between the headspace and sample volumes. Equation (1) is expressed in the form of Eq. (2) from which a graph of 1/A against β can be plotted, in which a and b are the slope and intercept, respectively. From the linear Eq. (2), the partition coefficient, K (equal to a/b) was calculated (Benjamin et al. 2011).

### Dynamic flavour release

An electronic nose (PEN 3, AIRSENSE Analytics GmbH, Schwerin, Germany) was used to measure the dynamic release of volatile flavour compounds with time. Approximately 5 mL emulsion was added to a 40 mL vial containing 2 mL of phosphate buffer (pH 3.5 / pH7) and heated in a bath at 40 °C for 10 min, then mixed with 0.004% volatile compound (nonanal/ethyl acetate, being the most and least hydrophobic of the four selected VOCs, respectively). The samples were transferred from the bath to a heating plate at 40 °C. A few seconds after starting the test, the emulsion was injected into the electronic nose at a uniform rate. Each measurement spanned for 100 s with 1 s sample intervals, a 200 s flush time and a 400 mL/min gas flow rate.

### Statistical analyses

All analyses were carried out in triplicate and the results expressed as mean ± standard deviation. The data were subjected to one-way analysis of variance (ANOVA) using the Statistical Package for Social Sciences (IBM SPSS Statistics for Windows, Version 26.0, Armonk, New York, USA). ANOVA and Duncan's multiple range tests were performed at a 95% confidence interval (p < 0.05) level to determine the significant difference between means.

## Results and discussion

### Emulsion characterization

**Fig. 1:**
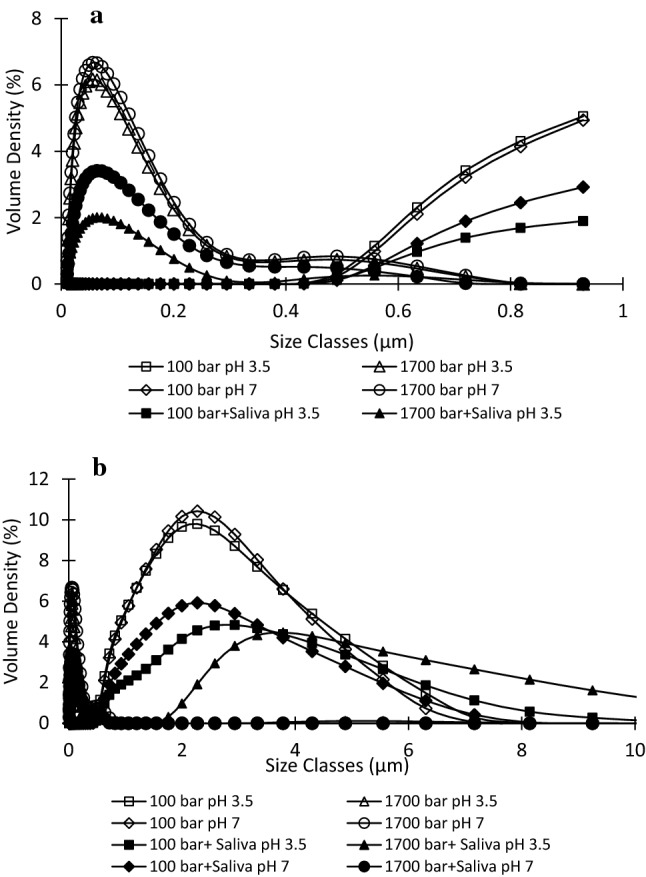
Particle size distribution of conventional and nano-emulsions at pH 3.5 and 7.0, with and without saliva addition: **a** distribution between 0 and 1 µm and **b** between 0 and 10 µm

Figure 1 shows the particle size distribution PSD of emulsions prepared at the lowest and highest homogenization pressures to illustrate the size and proportion of all particles present in conventional and nano-emulsions which could provide information on the behaviour and stability of the emulsions. The narrower peaks and smaller particle sizes of nano-emulsions indicated that the particles were uniformly distributed, implying better emulsion stability (Yang et al. 2018). Broader peaks with a higher volume density (%) observed at lower pressures for conventional and saliva-containing emulsions, especially at pH 3.5, indicated that these emulsions were of lower homogeneity and larger surface contact areas than nano-emulsions, making them prone to coalescence and flocculation (Yuliani et al. 2018). In saliva-containing emulsions at pH 3.5, two distinct peaks were observed at 1700 bar, the first between 0.03 and 0.40 µm and the second between 2–10 µm. Since this was not observed in saliva-containing emulsions at pH 7.0, the peak at larger particle size was attributed to saliva-induced flocculation due to the emulsion end pH (5.6) being close to the pI of β-lactoglobulin (≈ 5.2), resulting in larger aggregated particles compared to nano-emulsions with uniformly distributed fine particles.

At 1000 and 1700 bar, nano-emulsions with small particle size (d_3,2_ = 0.04–0.21 µm) were formed, including saliva-containing emulsions (Table 2). d_3,2_ provides information on particle size in relation to specific surface area and is more sensitive to the presence of fine droplets in the size distribution while d_4,3_ reflects the size of those particles constituting the bulk of the sample volume and is most sensitive to the large particles in the size distribution (Malvern Instruments 2015). As seen from Table 2, d4,3 showed strong effect (about 20 times higher) of saliva-induced flocculation on the droplet sizes. At high homogenization pressures, nano-emulsions had significantly (*p* < 0.05) smaller particle size than conventional emulsions. This implies improved stability because emulsions with smaller droplets are less susceptible to destabilization mechanisms such as flocculation and creaming (Yang et al. 2018).

**Table 2:** Emulsion characteristics at various homogenization pressures, pH 3.5 and 7.0, with and without saliva addition

Emulsion sample	d_3,2_ (µm)		d_4,3_ (µm)		Particle charge (mmole charge/litre)		Viscosity (mPas)	
	pH 3.5	pH 7.0	pH 3.5	pH 7.0	pH 3.5	pH 7.0	pH 3.5	pH 7.0
100 bar	1.67 ± 0.01^d^	1.66 ± 0.01^d^	2.32 ± 0.04^c^	2.27 ± 0.03^bc^	7.60 ± 0.28^ h^	− 5.13 ± 0.12^b^	0.93 ± 0.02^i^	0.55 ± 0.00^d^
100 bar + saliva	3.71 ± 0.37^g^	2.70 ± 0.04^f^	39.52 ± 3.02^ h^	33.24 ± 1.43^g^	− 0.67 ± 0.12^f^	− 4.73 ± 0.11^c^	1.48 ± 0.02^j^	0.54 ± 0.01^cd^
500 bar	0.66 ± 0.01^c^	0.66 ± 0.01^c^	0.77 ± 0.02^ab^	0.78 ± 0.01^ab^	7.73 ± 0.11^ h^	− 5.00 ± 0.00^b^	0.67 ± 0.01^g^	0.63 ± 0.02^e^
500 bar + saliva	1.97 ± 0.08^e^	1.62 ± 0.09^d^	28.53 ± 1.61^f^	31.69 ± 2.19^g^	− 0.73 ± 0.12^f^	− 4.40 ± 0.20^d^	1.83 ± 0.01^ k^	0.27 ± 0.01^a^
1000 bar	0.07 ± 0.00^a^	0.06 ± 0.00^a^	0.40 ± 0.17^a^	0.21 ± 0.01^a^	7.60 ± 0.00^ h^	− 5.47 ± 0.12^a^	0.53 ± 0.01^b^	0.66 ± 0.01^f^
1000 bar + saliva	0.21 ± 0.02^b^	0.20 ± 0.01^b^	28.25 ± 4.35^f^	25.83 ± 2.33^e^	− 2.06 ± 0.12^e^	− 4.33 ± 0.12^d^	3.32 ± 0.01^ l^	0.69 ± 0.02^g^
1700 bar	0.05 ± 0.00^a^	0.04 ± 0.00^a^	0.15 ± 0.01^a^	0.09 ± 0.00^a^	7.20 ± 0.00^ g^	− 5.53 ± 0.11^a^	0.53 ± 0.01^bc^	0.66 ± 0.01^f^
1700 bar + saliva	0.14 ± 0.01^ab^	0.09 ± 0.00^a^	20.63 ± 1.45^d^	21.48 ± 1.58^d^	− 2.20 ± 0.20^e^	− 4.40 ± 0.00^d^	3.76 ± 0.02^ m^	0.75 ± 0.06^ h^

Values are given as means ± standard deviation with sample size n = 3. Different superscripts between rows and columns of each parameter indicate significant differences between samples (p < 0.05)

A similar trend of smaller droplet sizes with increased homogenization pressure has been reported previously (Kwan and Davidov-Pardo 2018; Liang et al. 2012). There is a larger surface area for β-lactoglobulin to adsorb onto with smaller oil droplets, resulting in more exposed proteins and an increased absolute surface charge observed in the nano-emulsions compared to conventional emulsions (Table 2). Variation in homogenization pressure had a minimal influence (2–9%) on the particle charge of emulsions, except for saliva-containing emulsions at pH 3.5, where a 70% increase in charge was recorded from 100 to 1700 bar. As particle size decreased with increased homogenization pressure, the apparent viscosities (Table 2) of emulsions at a fixed shear rate (100/s) significantly increased from 1.48 mPas for 100 bar to 3.76 mPas for 1700 bar, in saliva-containing emulsions at pH 3.5 but remained below 1 mPas under other conditions. The viscosity of an oil-in-water emulsion system is dependent on the dispersed phase volume fraction or droplet concentration which increased as high-pressure homogenization broke apart the droplets (Yang et al. 2018). While high-pressure homogenization produces nano-sized droplets, it also increases the volume fraction of these smaller droplets in the emulsion system, consequently increasing the viscosity (Liang et al. 2012). Besides the higher volume of oil droplets and probable flocculation (at pH 3.5), increased viscosity in saliva-containing nano-emulsions could also be due to salivary viscosity (≈1.5 mPas), which is primarily caused by the glycoprotein mucin, which has a large molecular weight and can form networks and trapping water molecules (Gittings et al. 2015). The decreased viscosity from 0.93 to 0.56 mPas with increasing homogenization pressure observed in emulsions without saliva at pH 3.5 could be due to the stability of the nano-emulsions characterized by small particle sizes (Table 2). Additionally, at such a low pH value of 3.5, there is limited hydrophobic interaction between β-lactoglobulin, and oil droplets compared to adsorption at pH 7.0, which could also explain the different viscosity behaviours at these pH values (Kontopidis et al. 2004).

**Fig. 2:**
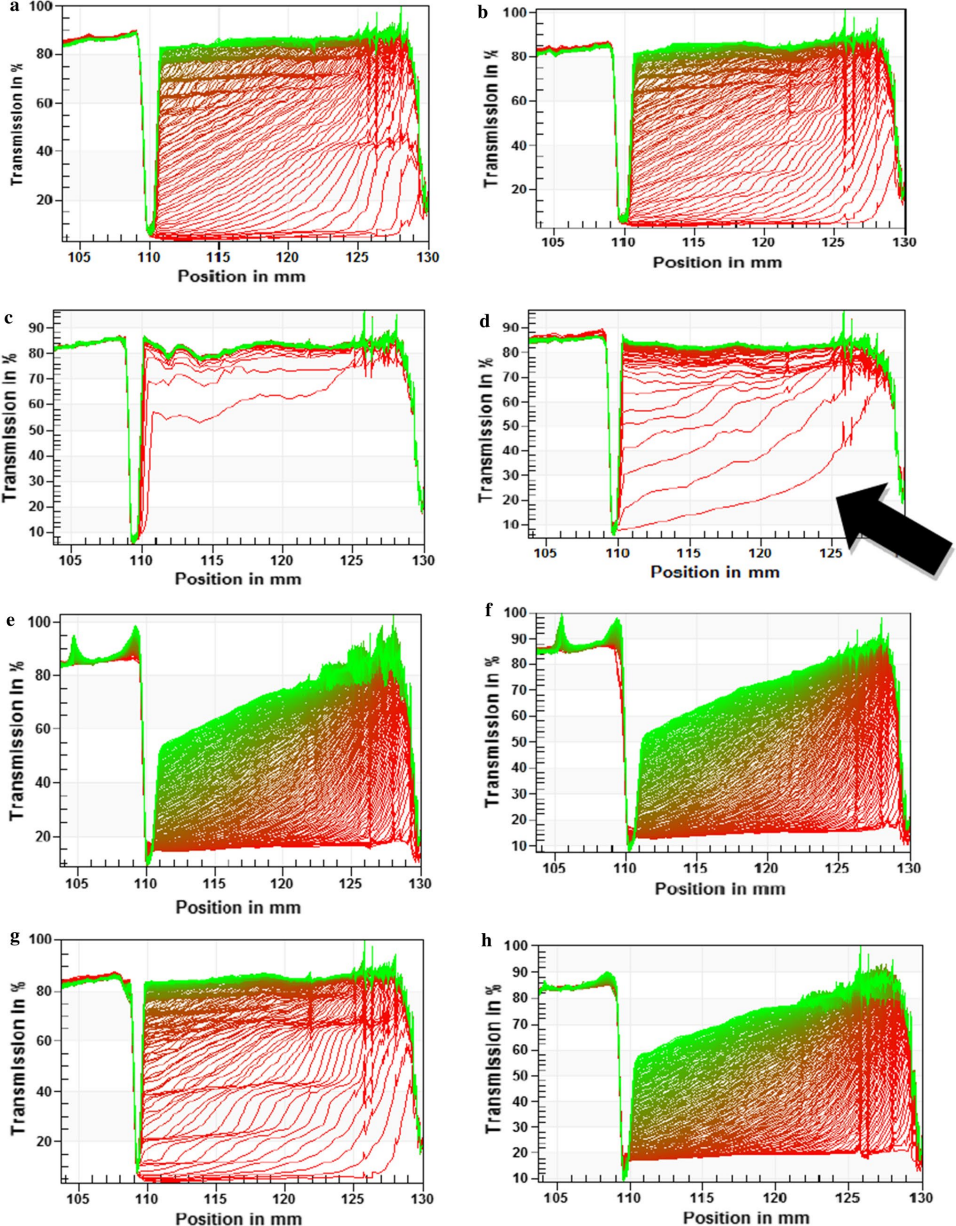
Transmission profiles during centrifugation of emulsions at **a** pH 3.5 100 bar, **b** pH 7.0 100 bar **c** pH 3.5 100 bar + saliva, **d** pH 7.0 100 bar + saliva, **e** pH 3.5 1700 bar, **f**) pH 7.0 1700 bar, **g** pH 3.5 1700 bar + saliva and **h** pH 7.0 1700 bar + saliva (Arrow indicates direction of creaming droplets from bottom to top of the tube)

Compared to conventional emulsions with an average creaming velocity (CV) of 24 µm/s, nano-emulsions at 1700 bar had a CV of 5 µm/s and better stability. CV and transmission profiles serve as measures of emulsion stability by the extent of phase separation during the application of centrifugal force. The light transmission profiles obtained from LUMiSizer (Fig. 2) reflect creaming as the droplets moved from the bottom of the tube (right side of the curve) to form a cream layer at the air–liquid phase boundary. The larger gaps between two consecutive transmission curves indicated that conventional emulsions at pH 3.5 and with saliva showed less stability against creaming. The intervals between consecutive transmission profiles gradually narrowed in nano-emulsions due to improved resistance to creaming caused by better emulsification of the nano-sized droplets and particle immobilization due to increased viscosity (Table 2). As confirmed by Liang et al. (2012), the observed decrease in creaming with increasing pressure was also due to reduced contact area of smaller droplets, which decreases the tendency of coalescence.

Saliva addition significantly increased d_3,2_ by a factor of 3 and d_4,3_ by a factor of 40 to 100 (Table 2) at all homogenization pressures. Significant differences in droplet size were recorded between saliva-containing emulsions prepared at pH 3.5 and pH 7.0. The particle size distribution of saliva showed an average of 50 μm (results not shown) which could be responsible for the larger particle sizes recorded in saliva-containing emulsions. For emulsions at pH 3.5, the particle charges (Table 2) ranged from 7.2 to 7.7 and − 2.2 to − 0.7 mol charge/litre in emulsions without and with saliva respectively, while emulsions at pH 7.0 had charges ranging from − 5.5 to − 5.0 and − 4.7 to − 4.3 mol charge/litre. Benjamin et al. (2013) reported in line with these observations a decrease in surface charge from 49 to 11 mV, increase in pH from 3.5 to 5 and droplet size from 0.2 to 30 µm on the addition of saliva to emulsions. The positive charge observed in non-saliva emulsions at pH 3.5 was due to its pH being below the pI of β-lactoglobulin (≈ 5.2). Saliva addition resulted in fewer charges in the emulsions at pH 3.5 and 7.0, with a more drastic reduction in saliva-containing emulsions at pH 3.5.

Kwan and Davidov-Pardo (2018) reported that saliva raises the pH of emulsions and weakens electrostatic repulsion, as seen by an increase in emulsion pH on saliva (pH 7.6) addition from 3.5 to 5.6 which is very close to the pI of β-lactoglobulin (≈ 5.2). At this pH, the net charge surrounding the particles is nearly zero (Table 2), resulting in less electrostatic repulsion, promoting droplet aggregation and, consequently, larger droplets formation than droplets at pH 7.0. This formation of relatively strong flocculate complexes between emulsion particles that are difficult to break by shearing could also account for the increased viscosity (Table 2) observed in saliva-containing emulsions at pH 3.5. The increased creaming is evidenced by wider gaps between horizontal and parallel sections in transmission profiles (Fig. 2c,d,g), which represent the creaming of polydisperse and aggregated droplets due to their varied velocities (Hoffmann 2016) as a function of their size.

Conventional saliva-containing emulsion at pH 3.5 and 100 bar showed the highest creaming velocity (CV) and were least stable, while nano-emulsion without saliva at pH 7.0 and 1700 bar had the lowest CV. At pH 3.5, saliva addition significantly increased the CV of conventional and nano-emulsions prepared at 100 and 1700 bar from 25 to 130 µm/s and 5 to 21 µm/s, respectively. There were no significant differences in CV between pH 3.5 and 7.0 except in saliva-containing emulsions at 100 bar where emulsion at pH 3.5 had a CV of 130 µm/s while 32 µm/s was recorded at pH 7.0. Previous research showed less flocculation and creaming in more negatively charged emulsions due to electrostatic repulsion preventing the close approach of the droplets (van Aken et al. 2007). Additionally, β-lactoglobulin shows better adsorption to the oil–water interface at pH 7.0 compared to pH 3.5, which improves the resistance of the emulsion system against phase separation (Paravisini and Guichard 2017).

### Flavour release from emulsion systems

The gas/liquid partition coefficients (K) at equilibrium were calculated using the PRV method to evaluate the effects of the VOC physicochemical properties and the interactions with other system components. K values of the VOCs were compared in emulsion systems with and without saliva, at varying pressures and pH 3.5 and 7.0 (Table 3). The partition coefficient of saliva-containing emulsions was studied only at pH 3.5 to evaluate the instability effect on flavour release since saliva raised the emulsion pH close to the pI of β-lactoglobulin.

**Table 3:** Comparison of partition coefficients (K)* of VOCs in conventional emulsions versus nano-emulsions at pH 3.5 and 7.0 with and without added saliva

Emulsion sample	Ethyl acetate		1-Penten-3-one		Trans-2-hexenal		Nonanal	
	pH 3.5	pH 7.0	pH 3.5	pH 7.0	pH 3.5	pH 7.0	pH 3.5	pH 7.0
100 bar	19.26 ± 10.99^b^	7.08 ± 0.82^a^	23.06 ± 6.56^f^	2.50 ± 0.69^d^	3.04 ± 1.81^ h^	1.66 ± 0.41^ g^	1.20 ± 0.1^ k^	1.18 ± 0.02^ k^
100 bar + saliva	22.95 ± 0.58^b^	n.d	13.13 ± 3.72^ef^	n.d	4.46 ± 2.68^hi^	n.d	1.83 ± 0.78^kl^	n.d
500 bar	n.d	7.11 ± 0.13^a^	n.d	2.42 ± 0.57^d^	n.d	1.60 ± 0.06^ g^	n.d	0.77 ± 0.02^jk^
500 bar + saliva	n.d	n.d	n.d	n.d	n.d	n.d	n.d	n.d
1000 bar	n.d	5.36 ± 1.18^a^	n.d	2.97 ± 0.68^d^	n.d	1.97 ± 0.07^gh^	n.d	0.75 ± 0.28^j^
1000 bar + saliva	n.d	n.d	n.d	n.d	n.d	n.d	n.d	n.d
1700 bar	21.81 ± 7.32^b^	5.42 ± 4.75^a^	6.08 ± 0.32^e^	2.53 ± 0.33^d^	2.29 ± 0.47^ h^	2.30 ± 0.07^ h^	1.39 ± 0.05^ k^	0.54 ± 0.29^j^
1700 bar + saliva	47.84 ± 8.55^c^	n.d	11.68 ± 3.79^e^	n.d	5.22 ± 2.78^i^	n.d	2.93 ± 0.62^ l^	n.d

Values expressed as mean ± standard deviation (X 10^–3^). Different superscripts between rows and columns of each VOC indicate significant differences between samples (p < 0.05)

The results show that the K values of ethyl acetate, the most volatile and hydrophilic compound, ranged from 19 × 10^−3^ to 48 × 10^–3^ and 5.4 × 10^−3^ to 7.1 × 10^–3^ at pH 3.5 and 7.0, respectively, while nonanal, the least volatile and most hydrophobic compound had K values ranging from 1 × 10^–3^ to 3 × 10^–3^ and 0.5 × 10^−3^ to 1.2 × 10^–3^ at pH 3.5 and 7.0, respectively. The release of volatile organic compounds from emulsion systems involves their diffusion through the lipid phase, interface, aqueous phase, and headspace. Reduction in partition coefficient implies a decrease in the concentration of the VOC in the headspace indicating better retention of that VOC in the emulsion. The partition coefficients (K) and dynamic flavour release were increased with decreasing hydrophobicity (log P) and increasing volatility (vapour pressure) of the flavour compounds (Table 1), release of ethyl acetate > 1-penten-3-one > trans-2-hexenal > nonanal. Ethyl acetate (log P = 0.7) showed higher K values at both pH 3.5 and 7.0 compared to nonanal (log P = 3.5) since its lower hydrophobicity enhances transfer from dispersed phase through a continuous phase to the headspace (Paravisini and Guichard 2017). More hydrophobic flavour compounds have been reported to be better retained in oil-in-water emulsion systems due to stronger hydrophobic interactions with the oil phase (Mao et al. 2017).

For emulsions prepared at pH 3.5, K was higher than for emulsions at pH 7.0. Change in pH affects the partition of flavour compounds, interactions between emulsifiers, VOCs, and other emulsion components (Mao et al. 2017). Emulsions at pH 7.0 showed better stability against creaming than at pH 3.5 (Fig. 2) and more uniform particle size distribution (Fig. 1) which implies that the VOCs remained better encapsulated in the emulsion system and thus decreased their transport to the gas phase. Benjamin et al. (2013) reported higher volatile release from unstable saliva-containing emulsions at pH 5.0 compared to more stable emulsions at pH 7.0. Similar behaviour of higher flavour release from unstable emulsions at low pH has been recorded (Ammari and Schroen 2018; Andriot et al. 1999; Jouenne and Crouzet 2000). Additionally, increased retention of hydrophobic compounds in emulsions at pH 7.0 compared to pH 3.5 (Table 3b) may be due to changes in the flexibility of β-lactoglobulin allowing better access to the primary and secondary hydrophobic sites (Guichard 2002). Previous authors similarly attributed better retention of VOCs on pH increase from 3.0 to 9.0 to flexibility of the protein molecules, surface exposure of residues, and the unfolding of peripheric α-helix and β-sheet (Jouenne and Crouzet 2000).

In emulsions at pH 3.5, there was no significant effect of homogenization pressure on partition coefficients of the investigated VOCs except in 1-penten-3-one (*without saliva*) and ethyl acetate (*with saliva*). Significantly lower K of 1-penten-3-one in nano-emulsion without saliva at pH 3.5 was observed. At 1700 bar, the emulsion was homogenous and stable allowing this VOC of intermediate hydrophobicity (log P = 1) to be better dispersed within the oil and water phases. In contrast to K values of conventional emulsions, a significant increase of K, around a factor of 2, in nano-emulsions at 1700 bar was observed in ethyl acetate, trans-2-hexenal and nonanal on saliva addition. Saliva addition leads to the "salting out" effect enhancing the diffusion of hydrophilic ethyl acetate to the gas phase (Mao et al. 2017; van Ruth et al. 2002). Emulsions with saliva showed less stability against creaming (Fig. 2) resulting in more oil phase transferred to the liquid–air surface on phase separation, which enhanced VOC release to the headspace since the oil phase is enriched with hydrophobic trans-2-hexenal and nonanal (Benjamin et al. 2013; Paravisini and Guichard 2017). In more stable emulsions at pH 7.0, increased homogenization pressure had a minor or no effect on the hydrophilic compounds and more on hydrophobic nonanal. Due to the change in oil volume, there is more surface area of stable oil droplets for nonanal to absorb into ensuing lower partition coefficient in nano-emulsion than conventional emulsion. The converse higher release of trans-2-hexenal in nano-emulsion may be attributed to its lower hydrophobicity than nonanal (Table 1); therefore, its release is less influenced by the larger surface area of oil droplets. Additionally, high-pressure treatments, as used for nano-emulsion preparation, lead to the unfolding of β-lactoglobulin and more exposure of its hydrophobic binding sites (Walker et al. 2004). Due to the increased surface hydrophobicity of the protein, it is hypothesized that trans-2-hexenal was not sufficiently hydrophobic to interact strongly with β-lactoglobulin, hence its increased release from nano-emulsions at pH 7.0.

**Fig. 3:**
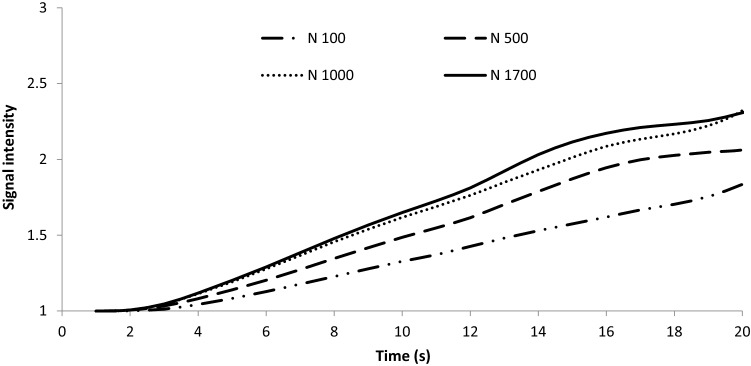
Dynamic flavour release curve for nonanal at pH 3.5 and different homogenization pressures measured by electronic nose

**Fig. 4:**
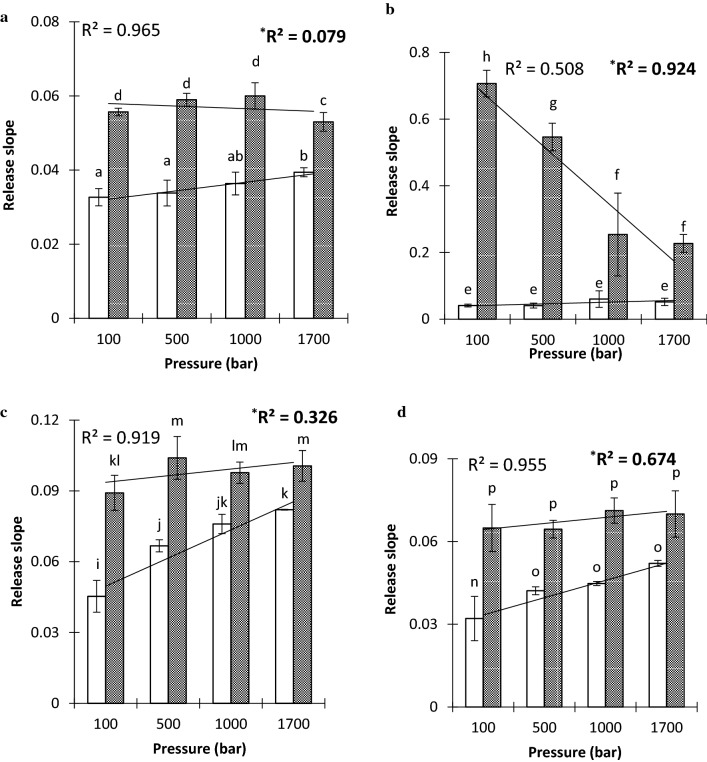
Dynamic flavour release slopes and correlation coefficient (R^2^) of **a** ethyl acetate at pH 3.5, **b** ethyl acetate at pH 7.0, **c** nonanal at pH 3.5 and **d** nonanal at pH 7.0 from conventional and nano-emulsions in the first 20 s of measurement. Different letters above bars indicate significant differences between samples. Error bars represent standard deviations. ^*^Filled bars and R.^2^ in the bold text represent saliva-containing emulsions

Dynamic release of the most hydrophilic and hydrophobic VOCs, ethyl acetate and nonanal, respectively, from emulsion systems were studied with an electronic nose resembling the first contact in the mouth (Fig. 3) Slopes from the first twenty seconds of measurements were calculated and are shown in Fig. 4. At pH 3.5, the dynamic release of ethyl acetate showed a strong correlation with homogenization pressure (R^2^ = 0.97) in emulsions without saliva, while a significant linear decrease in release (R^2^ = 0.92) with increased homogenization pressure was observed at pH 7.0 with saliva. The change in the distribution of the oil volume phase was likely to be the main cause of the increased VOC release. Nano-sized droplets have a larger total volume of oil which is an unsuitable environment for the hydrophilic ethyl acetate; thus, the VOC is forced to escape into the headspace (Guichard 2002). Conversely, higher VOC retention in nano-emulsion than conventional was observed in saliva-containing emulsions at pH 3.5 and 7.0. In the conventional emulsions, saliva induced flocculation, larger oil droplets (Table 2) and phase separation/creaming (Fig. 2) into two distinct flocculated oil and water phases, causing ethyl acetate to leave the unfavourable matrix (Benjamin et al. 2013). The higher homogeneity of the nano-emulsion particles (Fig. 1) and stability improved dissolution of hydrophilic ethyl acetate (log P = 0.7) in the saliva and water phase of the emulsion, consequently increasing the VOC retention. Increased viscosities of nano-emulsions (Table 2) may also have delayed mass transfer of the VOC at the emulsion–gas interface (Giroux et al. 2007). At pH 7.0, emulsion systems without saliva had better homogeneity and stability (Figs. 1, 4) compared to pH 3.5, which could explain why homogenization pressure had no significant effect on ethyl acetate release at this pH. The high release slope of ethyl acetate from saliva-containing emulsion (Fig. 3b) is related to high release of VOC at pH 7.0 due to its high vapour pressure and improved stability compared to pH 3.5.

Homogenization pressure was significantly correlated with nonanal release from non-saliva emulsions at pH 3.5 (R^2^ = 0.92) and pH 7.0 (R^2^ = 0.96) and indicated a greatly increased release of nonanal from nano-emulsions without saliva at pH 3.5 and 7.0 compared to conventional emulsions at low homogenization pressures. It was expected that high-pressure homogenization forms emulsions with more homogenized small fat droplets that attract hydrophobic nonanal (Paravisini and Guichard 2017), but there was a higher dynamic release because the flavoured emulsion had not achieved a state of equilibrium causing the hydrophobic nonanal to release from the oil-in-water emulsion with additional water from the buffer and saliva. In contrast, decreased release of nonanal from nano-emulsion was observed at pH 7.0 in static analysis under equilibrium due to the larger surface area of stable oil droplets enabling stronger binding of the hydrophobic VOC. Additionally, nano-sized oil droplets may lead to faster VOC mass transfer due to the increased interfacial area and shorter diffusion distance through the droplets (Mao et al. 2017).

## Conclusion

The structural stability of nano and conventional emulsions and the release of volatile organic compounds were analysed for preparation pH's of 3.5 and 7.0, with and without the addition of saliva. Whereas the addition of saliva did not affect emulsion pH for pH 7.0, for pH 3.5 it resulted in an increase in pH value to 5.6. The latter emulsions showed limited stability, which could be explained from the pH being close to the pI of β-lactoglobulin (5.2) used as an emulsifier. This caused the net surface charges being close to zero, resulting in flocculation, larger particles, and a higher creaming velocity than emulsions without saliva and at pH 7.0. Nano-emulsions were structurally more stable than conventional emulsions, with and without saliva.

Static headspace analysis showed in general significantly lower partition coefficients (K) of flavour compounds in emulsions at pH 7.0 than preparations at pH 3.5. Flavour release decreased with decreasing volatility and increasing hydrophobicity; ethyl acetate showed the highest K value for emulsions, whereas nonanal had the lowest value. Flavour compounds in saliva-containing emulsions were released to a greater extent due to aggregation and creaming with the consequent change in the oil volume phase distribution into a separated top layer enriched with hydrophobic volatile organic compounds. In comparison to static headspace analysis, where emulsion pH and physicochemical properties of volatile compounds had greater impacts on flavour release than homogenization pressures, dynamic analysis revealed more apparent differences in VOC release between conventional and nano-emulsions. This study also highlights the significance of emulsion stability on VOC and distinct release behaviours observed under static and dynamic conditions. High-pressure homogenized nano-emulsions can be used in food applications for beverages with higher stability and different flavour release perception than conventional emulsions.

## Data Availability

The datasets used and analyzed during the study are available from the corresponding author on reasonable request.
